# Serological and molecular positivity for *Leishmania infantum* in dogs referred to two veterinary hospitals in Barcelona, Spain

**DOI:** 10.1186/s13071-026-07545-4

**Published:** 2026-06-26

**Authors:** Paula Corominas, Mireia Fernández, Clàudia Viñeta, Carles Blasi-Brugué, Gloria Pol, Olga Francino, Xavier Roura

**Affiliations:** 1 Hospital Clínic Veterinari, Universitat Autònoma de Barcelona, Bellaterra, Spain; 2 AniCura Glòries Hospital Veterinari, Barcelona, Spain; 3 School of Veterinary Medicine, University College Dublin, Dublin, Ireland; 4 LETIPharma, Barcelona, Spain; 5 Servei Veterinari de Genètica Molecular, Facultat de Veterinària, Universitat Autònoma de Barcelona, Bellaterra, Spain

**Keywords:** Canine, Leishmaniosis, Urban, Peri-urban, Serology, qPCR

## Abstract

**Background:**

*Leishmania infantum* is endemic in the Mediterranean basin, with dogs serving as the primary domestic reservoir for human infection. Although the city of Barcelona has historically been considered a low-risk area, recent environmental and demographic changes may favor parasite transmission. This study aimed to estimate and compare the positivity to *L. infantum* using serological and molecular methods in dogs referred to a veterinary hospital in the city of Barcelona (urban) and to another located in its residential metropolitan area (peri- urban), and to assess potential associated risk factors.

**Methods:**

A cross-sectional study was conducted from September 2022 to September 2023 at two veterinary referral hospitals in the urban (hospital A) and peri-urban (hospital B) areas of Barcelona. Blood and conjunctival swab samples were analyzed by quantitative ELISA and qPCR targeting *L. infantum* kinetoplast DNA.

**Results:**

A total of 189 client-owned dogs were enrolled (77 from hospital A and 112 from hospital B). Of these, 93/189 (49.2%) were presented for an annual check-up and appeared healthy, while 96/189 (50.8%) were sick. *Leishmania infantum* infection—defined as positivity by ELISA and/or qPCR—was detected in 10.6% (20/189) dogs, exclusively among sick patients (20/96;20.8%). None of the healthy dogs tested positive. Both serological and molecular tests yielded a 6.3% (12/189) positivity. All qPCR-positive dogs belonged to hospital B, resulting in a higher positivity rate among dogs referred to the peri-urban hospital (15.2%) than among those referred to the urban hospital (3.9%). Significant associations were identified between positivity and both clinical status (*P* < 0.001) and hospital location (*P* = 0.013), but not with the other variables evaluated.

**Conclusion:**

The findings confirm active *L. infantum* transmission among dogs in Barcelona, with higher positivity among dogs referred to a hospital in the peri-urban area.

**Graphical Abstract:**

**Figure:**
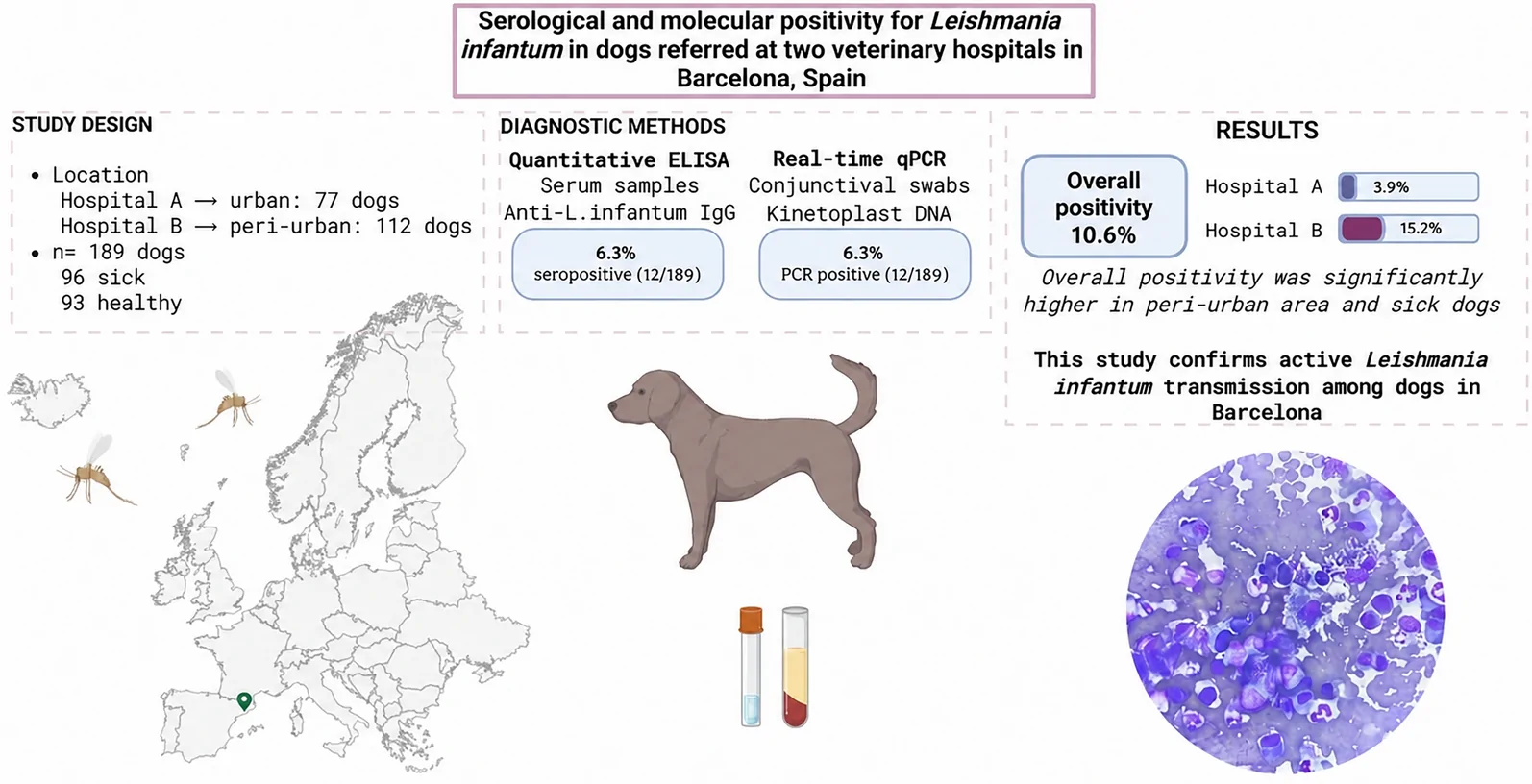

## Background

Leishmaniosis caused by *Leishmania infantum* is a vector-borne zoonotic disease of major concern in both human and veterinary medicine, transmitted through the bite of infected phlebotomine sand flies [1]. It is classified by the World Health Organization (WHO) as a neglected tropical disease, a group of chronic, poverty-associated conditions that affect both humans and animals, impose a substantial burden on public and animal health, and often involve complex transmission cycles in which animals act as key reservoirs [2].

In southern Europe, particularly in the Mediterranean basin, *L. infantum* is the main causative agent of both human and canine leishmaniosis (CanL). Domestic dogs are the principal reservoir, although other wild and synanthropic hosts may contribute to parasite maintenance in the environment [1, 3–6]. Disease prevalence varies according to climate, vector and reservoir host dynamics, and human behavior and movement patterns, with the annual activity cycle of sand flies representing a key driver [3, 4, 7]. Therefore, the risk of human infection is closely linked to the prevalence of infection in the canine population and the density of infected sand fly vectors [6–9].

Recent studies have reported autochthonous human and canine cases in areas previously considered non-endemic, including urban environments, in association with factors such as increased movement of people and domestic dogs, climatic changes favoring the presence and expansion of sand fly vectors, the identification of additional competent reservoirs and vectors, and improved surveillance [8, 10–12, 14]. In Spain, both human and canine leishmaniosis have shown increasing incidence [3, 9, 11, 15]. In addition, alternative reservoirs, including lagomorphs and urban rodents, have been identified, indicating variation in transmission dynamics [5, 16–18].

These findings raise concerns about the urbanization of leishmaniosis and its adaptation to new ecological niches, challenging the traditional view that CanL is restricted to peri-urban and rural environments.

The present study aimed to estimate and compare *L. infantum* positivity, as determined by serological and molecular techniques, in dogs referred to two veterinary hospitals, one located in the city of Barcelona (urban) and the other in its residential metropolitan area (peri-urban). In addition, the study investigated and compared potential risk factors associated with positivity.

## Methods

A prospective, cross-sectional, multicenter study was conducted from September 2022 to September 2023 at two veterinary referral hospitals in the province of Barcelona, Spain. AniCura Glòries Hospital Veterinari (hospital A) was in the city of Barcelona (urban area), and Hospital Clínic Veterinari at the Universitat Autònoma de Barcelona (hospital B) was situated 20 km from Barcelona city (peri-urban area). Client-owned dogs presenting either for routine health checks or with clinical signs compatible with leishmaniosis were enrolled and were classified as apparently healthy or clinically sick based on the attending clinician’s assessment at presentation, respectively. The target enrollment was approximately 10 dogs per month at each hospital, based on feasibility and expected clinical caseload, with about half classified as healthy and half as sick. Dogs were eligible for inclusion if they lived exclusively in the city of Barcelona (hospital A) or exclusively in a peri-urban area (hospital B), defined by a mixture of forested, cultivated land, and residential area around Barcelona city. Dogs previously diagnosed with leishmaniosis or with a history of travel to other leishmaniosis-endemic areas were excluded. Serum samples and conjunctival swabs were collected for serological and molecular analyses, respectively.

Data recorded for each dog included hospital of origin, clinical status, sex, neuter status, breed, age (grouped into 6 months to 5 years, 5 to 9 years, or > 9 years), body weight (categorized as < 5 kg, 5–15 kg, 15–25 kg, or > 25 kg), coat length, sampling season, cohabitation with other dogs, and the use and adequacy of preventive measures against leishmaniosis. In sick dogs, organ involvement and duration of disease were also recorded.

Blood samples were collected by standard venipuncture, and serum was obtained after clot formation. Anti-*L. infantum* IgG antibodies were quantified using a commercial ELISA (Ingezim® Leishmania VET, Gold Standard, Madrid, Spain), with results interpreted according to the reference values provided by LETILab (LETI Pharma, Barcelona, Spain) [19]. Antibody levels were further categorized into five groups based on their equivalence (Eq.) to the immunofluorescence assay (IFA) titers: doubtful (26–35%, Eq. IFA 1/80), low (36–65%, Eq. IFA 1/160), medium (66–90%, Eq. IFA 1/320), high (91–135%, Eq. IFA 1/640), and very high (≥ 136%, Eq. IFA ≥ 1/1280).

Conjunctival swabs were collected from both eyes using sterile cotton swabs and stored under refrigeration until processing. Detection of *L. infantum* DNA was performed by real-time quantitative polymerase chain reaction (qPCR) targeting kinetoplast DNA, as previously described [20]. Parasite load was categorized according to delta Ct values between *Leishmania* Ct and the endogenous control 18S rRNA Ct, as low (> 15), medium (5 to 15), or high (< 5).

Data were analyzed using IBM SPSS Statistics (version 29; IBM Corp., Armonk, NY, USA). Categorical variables were expressed as percentages, and continuous variables as mean ± SD or median (range), depending on distribution. Associations between categorical variables and serological positivity, qPCR positivity, and overall positivity were assessed using Chi-square or Fisher’s exact tests, with statistical significance set at *P* < 0.05.

## Results

### Study population

The characteristics of the study population are summarized in Table 1.

**Table 1:** Data recorded from the study population

Study population	Hospital A (urban)	Hospital B (peri-urban)
Total = 189	77/189 (40.7%)	112/189 (59.3%)
Clinical status		
Healthy 93/189 (49.2%)	41/93 (44.1%)	52/93 (55.9%)
Sick 96/189 (50.8%)	36/96 (37.5%)	60/96 (62.5%)
Sex		
Male 110/189 (58.2%)	44/77 (57.1%)	66/112 (58.9%)
Female 78/189 (41.3%)	32/77 (41.6%)	46/112 (41.1%)
Not recorded 1/189 (0.5%)	1/77 (1.3%)	0/112 (0.0%)
Neuter status		
Entire 73/189 (38.6%)	Male/Female	Male/Female
Neutered 115/189 (60.8%)	22/44 (50%)/12/32 (37.5%)	22/66 (33.3%)/17/46 (37.0%)
Not recorded 1/189 (0.5%)	22/44 (50%)/20/32 (62.5%)	44/66 (66.7%)/29/46 (63.0%)
Breed (most common)		
	Crossbred 23/77 (29.9%)	Crossbred 22/112 (10.7%)
	Golden retriever 4/77 (5.2%)	Labrador retriever 12/112 (10.7%)
	Yorkshire terrier 4/77 (5.2%)	Golden retriever 8/112 (7.1%)
	Labrador retriever 3/77 (3.9%)	American Staffordshire 5/112 (4.5%)
	American Staffordshire 3/77 (3.9%)	American bully 5/112 (4.5%)
Age		
< 5 years 46/189 (24.3%)	21/77 (27.3%)	25/112 (22.3%)
5–9 years 50/189 (26.5%)	19/77 (24.7%)	31/112 (27.7%)
> 9 years 91/189 (48.1%)	36/77 (46.8%)	55/112 (49.1%)
Not recorded 2/189 (1.1%)	1/77 (1.3%)	1/112 (0.9%)
Body weight		
< 5 kg 16/189 (8.5%)	9/77 (11.7%)	7/112 (6.3%)
5–15 kg 51/189 (27.0%)	25/77 (32.5%)	26/112 (23.2%)
15–25 kg 43/189 (22.8%)	14/77 (18.2%)	29/112 (25.9%)
> 25 kg 71/189 (37.6%)	28/77 (36.4%)	43/112 (38.4%)
Not recorded 8/189 (4.2%)	1/77 (1.3%)	7/112 (6.3%)
Coat length		
Short 100/189 (52.9%)	40/77 (51.9%)	60/112 (53.6%)
Medium 39/189 (20.6%)	14/77 (18.2%)	25/112 (22.3%)
Long 37/189 (19.6%)	10/77 (13.0%)	27/112 (24.1%)
Not recorded 13/189 (6.9%)	13/77 (16.9%)	0/112 (0.0%)
Sampling season		
Spring 46/189 (24.3%)	28/77 (36.4%)	18/112 (16.1%)
Summer 31/189 (16.4%)	7/77 (9.1%)	24/112 (21.4%)
Autumn 65/189 (34.4%)	35/77 (45.5%)	30/112 (26.8%)
Winter 47/189 (24.9%)	7/77 (9.1%)	40/112 (35.7%)
Cohabitation with other dogs		
Yes 48/189 (25.4%)	17/77 (22.1%)	31/112 (27.7%)
No 119/189 (63.0%)	48/77 (62.3%)	71/112 (63.4%)
Not recorded 22/189 (11.6%)	12/77 (15.6%)	10/112 (8.9%)
Use of preventive measures^a^		
Yes 131/159 (82.4%)	47/59 (79.7%)	84/100 (84.0%)
No 25/159 (17.6%)	12/59 (20.3%)	16/100 (16.0%)
Not recorded 30/189 (15.9%)	18/77 (23.4%)	12/112 (10.7%)
Adequacy of preventive measures		
Yes 35/131 (26.7%)	6/47 (12.8%)	29/84 (34.5%)
No 96/131 (73.3%)	41/47 (87.2%)	55/84 (65.5%)
Specific product for prevention^b^		
Collar 24/77 (31.2%)	6/40 (15.0%)	18/37 (48.6%)
Spot-on 17/77 (22.1%)	9/ 40 (22.5%)	8/37 (21.6%)
Vaccine 5/77 (6.5%)	0/40 (0.0%)	5/37 (13.5%)
Tablet 2/77 (2.6%)	0/40 (0.0%)	2/37 (5.4%)
Spray 1/77 (1.3%)	1/40 (2.5%)	0/37 (0.0%)
Combination 28/77 (36.4%)	24/40 (60.0%)	4/37 (10.8%)
Unknown 54/131 (41.2%)	7/47 (14.9%)	47/84 (56.0%)
Organ involvement (sick dogs)		
Gastrointestinal 25/96 (26.0%)	11/36 (30.6%)	14/60 (23.3%)
Urinary/renal 11/96 (11.5%)	2/36 (5.6%)	9/60 (15.0%)
Respiratory 10/96 (10.4%)	2/36 (5.6%)	8/60 (13.3%)
Hematologic 9/96 (9.4%)	2/36 (5.6%)	7/60 (11.7%)
Orthopedic 8/96 (8.3%)	5/36 (13.95)	3/60 (5.0%)
Dermatologic 5/96 (5.2%)	3/36 (8.3%)	2/60 (3.3%)
Ocular 1/96 (1.0%)	1/36 (2.8%)	0/60 (0.0%)
Multiple 27/96 (28.1%)	10/36 (27.8%)	17/60 (28.3%)
Disease duration (sick dogs)		
Acute 25/96 (26.0%)	6/36 (16.7%)	19/60 (31.7%)
Subacute 18/96 (18.8%)	11/36 (30.6%)	7/60 (11.7%)
Chronic 53/96 (55.2%)	19/36 (52.8%)	34/60 (56.7%)

^a^Information about preventive measures against sand fly bites was reported in 159/189 (84.1%) dogs

^b^Information regarding specific product for prevention was available in 77/131 (58.8%) dogs in which preventive measures were implemented

### Positivity and association with diagnostic tests used

The overall positivity, defined as positivity by either qPCR and/or serology, was 10.6% (20/189). All positive cases were clinically sick dogs, corresponding to a positivity of 20.8% (20/96). Positivity in hospital A (urban) was 3.9% (3/77), whereas positivity in hospital B (peri-urban) was 15.2% (17/112). The seasonal distribution of positive dogs was as follows: autumn 10/20 (50.0%), winter 7/20 (35.0%), spring 2/20 (10.0%), and summer 1/20 (5.0%).

Serology was positive in 12/189 (6.3%) dogs, with 3/12 (25.0%) from hospital A and 9/12 (75.0%) from hospital B. Circulating *Leishmania*-specific antibody levels were distributed as follows: doubtful (2/12; 1 per hospital), low (2/12; 1 per hospital), medium (2/12; both from hospital B), high (3/12; 1 from hospital A and 2 from hospital B), and very high (3/12; all from hospital B).

qPCR was positive in 12/189 (6.3%) dogs, all of them from hospital B. Parasite load was categorized as low in 7/12 (58.3%), medium in 4/12 (33.3%), and high in 1/12 (8.3%). Four qPCR-positive dogs were also seropositive, with high (1/4) or very high (3/4) anti-*Leishmania* antibody levels (Table 2).

**Table 2:** Diagnostic results and distribution of positive dogs between the two hospitals

Patient	Hospital (A/B)	Clinical status (healthy/sick)	qPCR ± (parasite load)	Serology ± (antibody amount)
1	B	Sick	+ (low)	-
2	B	Sick	+ (low)	-
3	B	Sick	+ (low)	-
4	B	Sick	+ (low)	-
5	B	Sick	+ (low)	-
6	B	Sick	+ (low)	-
7	B	Sick	+ (medium)	-
8	B	Sick	+ (medium)	-
9	B	Sick	+ (low)	+ (high)
10	B	Sick	+ (medium)	+ (very high)
11	B	Sick	+ (medium)	+ (very high)
12	B	Sick	+ (high)	+ (very high)
13	B	Sick	-	+ (doubt)
14	B	Sick	-	+ (low)
15	B	Sick	-	+ (medium)
16	B	Sick	-	+ (medium)
17	B	Sick	-	+ (high)
18	A	Sick	-	+ (doubt)
19	A	Sick	-	+ (low)
20	A	Sick	-	+ (high)

### Positivity and association with epidemiological variables

Interpretation of several comparisons was limited by small subgroup sizes, which reduced statistical power and the validity of χ^2^ assumptions. Nevertheless, overall positivity was significantly higher in sick than in healthy dogs (χ^2^ = 21.668, *df* = 1, *P* < 0.001) and in the peri-urban hospital B than in the urban hospital A (χ^2^ = 6.12, *df* = 1, *P* = 0.013). No significant associations were found with season, sex, neuter status, body weight category, breed, age group, coat length, presence of other dogs in the household, type or duration of clinical signs, or the use, type, mode, and frequency of preventive measures (all *P* > 0.05).

## Discussion

This study provides hospital-based data on *L. infantum* infection in dogs referred to two veterinary hospitals in Barcelona. The overall positivity of 10.6% was consistent with previous reports from the same area, where estimates for canine leishmaniosis range from 0.5% to 16.7% [15, 21], and with the general seroprevalence reported in Catalonia (7.5–13.5%) [13, 22], although direct comparisons are limited by differences in study design and sampled populations. In addition, estimates within Catalonia vary substantially according to province, diagnostic methodology, and study population, with reported values of 14.5–16.7% in Barcelona [21], 19.5–24.6% in Girona [23], approximately 33% in Lleida [24], and up to 51.7% in Tarragona [25]. The positivity observed here was also lower than that reported in other endemic regions of Spain, including the Balearic Islands, Valencia, and southern areas [13, 21, 22].

A clear geographical gradient was identified, with significantly higher positivity in dogs referred to the peri-urban hospital than in those referred to the urban hospital. However, these differences should be interpreted with caution, as they may also reflect variations in referral patterns, case selection, and hospital populations rather than true differences in infection pressure. Similar differences between urban and peri-urban or rural settings have been reported previously in Spain and other endemic regions. In Spain, Gálvez et al. [21] found higher seroprevalence in rural (60%) and peri-urban (29%) habitats than in urban habitats (11%), although no statistically significant association between habitat and *L. infantum* infection was demonstrated in that study. Similarly, De Almeida Braz et al. [26], using serology and qPCR, reported prevalence rates of 26.6% in rural areas and 10.6% in urban areas of Mãe D’Água, Brazil. In Spain, these differences between urban and peri-urban or rural areas may be explained, at least in part, by the ecology of *P. perniciosus*, the main vector in the Iberian Peninsula, which thrives in environments with vegetation, organic matter, and suitable resting and breeding sites [4]. Nevertheless, although urban settings are generally less favorable for vector development, sand flies have been detected in city parks and peridomestic environments in Barcelona [18], confirming that transmission may also occur in urban areas. Moreover, the detection of *L. infantum* DNA in Norway rats from sewers and public parks [17, 18] suggests that urban reservoirs other than dogs could contribute to parasite maintenance. This reinforces the need to consider multiple host species when assessing zoonotic risk and when investigating the mechanisms underlying potential leishmaniosis outbreaks within a One Health framework integrating animal, human, and environmental factors [5, 8, 27, 28].

All infected dogs in this study were clinically sick, and no apparently healthy carriers were identified. This contrasts with previous reports from other endemic regions in which subclinical infection is common [22, 23, 29, 30]. Several factors may account for this discrepancy. First, although both sick and healthy dogs were included to better reflect the epidemiological scenario of an endemic area, the study population consisted of client-owned dogs receiving regular veterinary care, which may represent a lower-risk group than stray or shelter dogs. Second, preventive measures against sand fly bites were reportedly used in a high proportion of dogs, although information on the correctness of application was largely unavailable; indeed, no statistically significant association was found between overall positivity and the use of preventive measures. Third, vector density and infection pressure in urban and peri-urban areas of Barcelona may be lower than in more intensely endemic regions. In addition, the relatively small sample size may have reduced the likelihood of detecting infection in dogs without clinical signs. The fact that all positive dogs were clinically sick, together with the higher positivity observed in the peri-urban setting, further highlights the importance of the sampling frame. Finally, referral hospital populations may differ from the general canine population in their case mix, with a greater proportion of dogs affected by chronic, complex, or multisystemic disorders, thereby increasing the likelihood of identifying infected animals.

Although most positive dogs were detected in autumn and winter, no statistically significant seasonal association was identified. One possible explanation for this pattern is that sand fly activity in Mediterranean regions typically peaks in late spring and summer, whereas clinical signs may not become apparent until weeks or months after infection. However, the uneven and relatively small number of dogs sampled across seasons limits the interpretation of these findings. Longitudinal studies incorporating entomological surveillance are therefore needed to clarify seasonal transmission dynamics in urban and peri-urban environments.

No significant associations were identified between positivity and sex, neuter status, age, body weight, breed, coat length, or cohabitation with other dogs. This agrees with some previous studies [21, 22, 31], but differs from others reporting increased risk in male, adult or geriatric, large, short-haired, or breed-associated populations [12, 23, 29–33]. The absence of significant associations for these variables may be related to the limited sample size, the predominance of client-owned dogs in the study population, and the spatial distribution of the sampled dogs.

Preventive measures were reported by most owners, but no protective effect was identified, as no statistically significant association was found between overall positivity and preventive measure use. This differs from previous studies [26, 33], in which preventive measures were associated with a reduced risk of infection. However, these findings should be interpreted cautiously, since self-reported preventive use does not guarantee correct application, adequate duration of efficacy, or product effectiveness against local vector populations. Notably, 13 of the 20 positive dogs identified in this study either had not received preventive measures or had not received them according to current recommendations. Although the number of infected dogs was too small to confirm a statistically significant association, these findings underscore the importance of correct and guideline-based use of preventive measures in dogs [8, 28, 34, 35].

The clinical profile of infected dogs in this study was dominated by chronic disease with multisystemic manifestations, which is consistent with the heterogeneous clinical spectrum of canine leishmaniosis described in the literature [3, 4, 34–36]. However, the relatively small number of infected dogs limited the ability to assess whether *Leishmania* infection was associated with any specific clinical presentation.

This study has several limitations. First, a non-random selection of approximately 10 dogs per month was performed, with approximately 50% classified as healthy and 50% as sick, which may have introduced selection bias, as the populations attending the two hospitals could have differed in health status and case mix. Therefore, the results reflect positivity within a clinical population and should not be interpreted as representative of the general canine population. Second, the relatively small number of positive dogs reduced statistical power, particularly for subgroup comparisons and multivariable analyses. Third, some variables included categories with few observations, limiting the validity of Chi-square tests. Finally, only conjunctival swabs were used for qPCR to minimize invasiveness, whereas the inclusion of additional tissues such as skin, bone marrow, or lymph nodes might have increased diagnostic sensitivity.

## Conclusions

This study provides hospital-based evidence of spatial heterogeneity in canine *L. infantum* infection in Barcelona, with higher positivity in the peri-urban hospital than in the urban hospital. These findings highlight differences in transmission patterns between urban and peri-urban settings, and support geographically stratified surveillance and context-specific prevention strategies for canine leishmaniosis in endemic areas. Integrated One Health approaches, incorporating vector ecology and potential alternative reservoirs, may further improve control strategies in increasingly urbanized environments.

## Data Availability

Data supporting the conclusions of this study are included in the manuscript.

## Abbreviations

CanL: Canine leishmaniosis
DNA: Deoxyribonucleic acid
ELISA: Enzyme-linked immunosorbent assay
Eq.: Equivalence
IFA: Immunofluorescence assay
IgG: Immunoglobulin G
*L. infantum*: *Leishmania infantum*
*P. perniciosus*: *Phlebotomus perniciosus*
qPCR: Quantitative polymerase chain reaction
RNA: Ribonucleic acid
WHO: World Health Organization
